# iCDA-CGR: Identification of circRNA-disease associations based on Chaos Game Representation

**DOI:** 10.1371/journal.pcbi.1007872

**Published:** 2020-05-18

**Authors:** Kai Zheng, Zhu-Hong You, Jian-Qiang Li, Lei Wang, Zhen-Hao Guo, Yu-An Huang

**Affiliations:** 1 School of Computer Science and Engineering, Central South University, Changsha, China; 2 Xinjiang Technical Institutes of Physics and Chemistry, Chinese Academy of Sciences, Urumqi, China; 3 College of Computer and Software Engineering, Shenzhen University, Shenzhen, China; 4 College of Information Science and Engineering, Zaozhuang University, Zaozhuang, China; 5 Department of Computing, Hong Kong Polytechnic University, Hung Hom, Hong Kong, China; Utrecht University, NETHERLANDS

## Abstract

Found in recent research, tumor cell invasion, proliferation, or other biological processes are controlled by circular RNA. Understanding the association between circRNAs and diseases is an important way to explore the pathogenesis of complex diseases and promote disease-targeted therapy. Most methods, such as *k*-mer and *PSSM*, based on the analysis of high-throughput expression data have the tendency to think functionally similar nucleic acid lack direct linear homology regardless of positional information and only quantify nonlinear sequence relationships. However, in many complex diseases, the sequence nonlinear relationship between the pathogenic nucleic acid and ordinary nucleic acid is not much different. Therefore, the analysis of positional information expression can help to predict the complex associations between circRNA and disease. To fill up this gap, we propose a new method, named iCDA-CGR, to predict the circRNA-disease associations. In particular, we introduce circRNA sequence information and quantifies the sequence nonlinear relationship of circRNA by Chaos Game Representation (CGR) technology based on the biological sequence position information for the first time in the circRNA-disease prediction model. In the cross-validation experiment, our method achieved 0.8533 AUC, which was significantly higher than other existing methods. In the validation of independent data sets including circ2Disease, circRNADisease and CRDD, the prediction accuracy of iCDA-CGR reached 95.18%, 90.64% and 95.89%. Moreover, in the case studies, 19 of the top 30 circRNA-disease associations predicted by iCDA-CGR on circRDisease dataset were confirmed by newly published literature. These results demonstrated that iCDA-CGR has outstanding robustness and stability, and can provide highly credible candidates for biological experiments.

## Author summary

Understanding the association between circRNAs and diseases is an important step to explore the pathogenesis of complex diseases and promote disease-targeted therapy. Computational methods contribute to discovering the potential disease-related circRNAs. Based on the analysis of the location information expression of biological sequences, the model of iCDA-CGR is proposed to predict the circRNA-disease associations by integrates multi-source information, including circRNA sequence information, gene-circRNA associations information, circRNA-disease associations information and the disease semantic information. In particular, the location information of circRNA sequences was first introduced into the circRNA-disease associations prediction model. The promising results on cross-validation and independent data sets demonstrated the effectiveness of the proposed model. We further implemented case studies, and 19 of the top 30 predicted scores of the proposed model were confirmed by recent experimental reports. The results show that iCDA-CGR model can effectively predict the potential circRNA-disease associations and provide highly reliable candidates for biological experiments, thus helping to further understand the complex disease mechanism.

## Introduction

Circular RNA (circRNA) is a type of non-coding RNA without 5' end caps or a 3' end poly (A) tails [1]. Since the discovery of circular RNA (circRNA) in RNA viruses 40 years ago, more than 100,000 circRNAs have been found in cells [2]. With the rapid development of RNA sequencing (RNA-seq) technology and bioinformatics, more and more studies have shown that circRNA plays an important role in many cell activities including effecting on arteriosclerosis, involving in the regulation of mRNA expression and regulating alternative splicing [3–8]. In addition, some evidence suggests that some diseases may be related to abnormal expression of circRNA. Zhou et al. found miR-141 is suppressed by circRNA_010567 through targeting TGF-beta1 to promote myocardial fibrosis[9]. Meanwhile, Liang *et al*. discovered that breast cancer proliferation and progression can be promoted by circ-ABCB10 through sponging miR-1271 [10]. Many scholars believe that many circRNAs can be used as tumor markers and therapeutic targets in clinical applications [11]. Based on the above reasons, confirming the potential association has gradually become a research hotspot in recent years. However, the high experimental cost and long experimental circle restrict the traditional experimental methods from verifying the association between circRNA and diseases on a large scale. In order to solve this problem, the calculation method rises in response to the proper time and conditions[12–16].

In recent years, in order to unify the standards of circRNAs obtained by experiment, many databases were established as circBase, CIRCpedia, deepBase, CircNet and circRNADb [17–21]. These databases provided biological essential information about circRNA, such as sequencing data and gene target. What’s more, there are many databases that choose to collect circRNAs that have been shown to be associated with various diseases, including CircR2Disease, circRNADisease, circFunBase, and Circ2Disease [22–25]. These databases provide data support for selecting candidates of potential circRNA-disease associations by computational methods. For example, Xiao *et al*. proposed a weighted dual-manifold regularized-based calculation model named MRLDC which integrates geometric information and intrinsic diversity of circRNA and disease feature spaces [26]. Although this method has achieved good results, there are only 331 association for training model. A small number of training samples may lead to insufficient robustness of the model. In addition, MRLDC only describes the behavior information in circRNA-disease association network, and cannot directly and accurately measure circRNA similarity and disease similarity from the attributes of circRNA and disease. Fan *et al*. proposed a computational model of KATZ measures for human circRNA-disease association prediction (KATZHCDA) using a heterogeneous network [27]. Similarly, this model also does not have enough training samples. Among them, 275 circRNAs, 36 diseases, and 312 associations were used. Although KATZHCDA uses circRNA expression profile information, its performance is still limited. Compared with the above two models, GHICD and RWRHCD have relatively sufficient training samples. They used 541 circRNAs, 83 diseases, and 592 associations[28]. It is worth noting that although they have achieved some effects and used the circRNA-gene association network to describe the attribute information of circRNA, the accuracy is still limited because the association network formed by circRNA and genes is very sparse.

**Fig 1:**
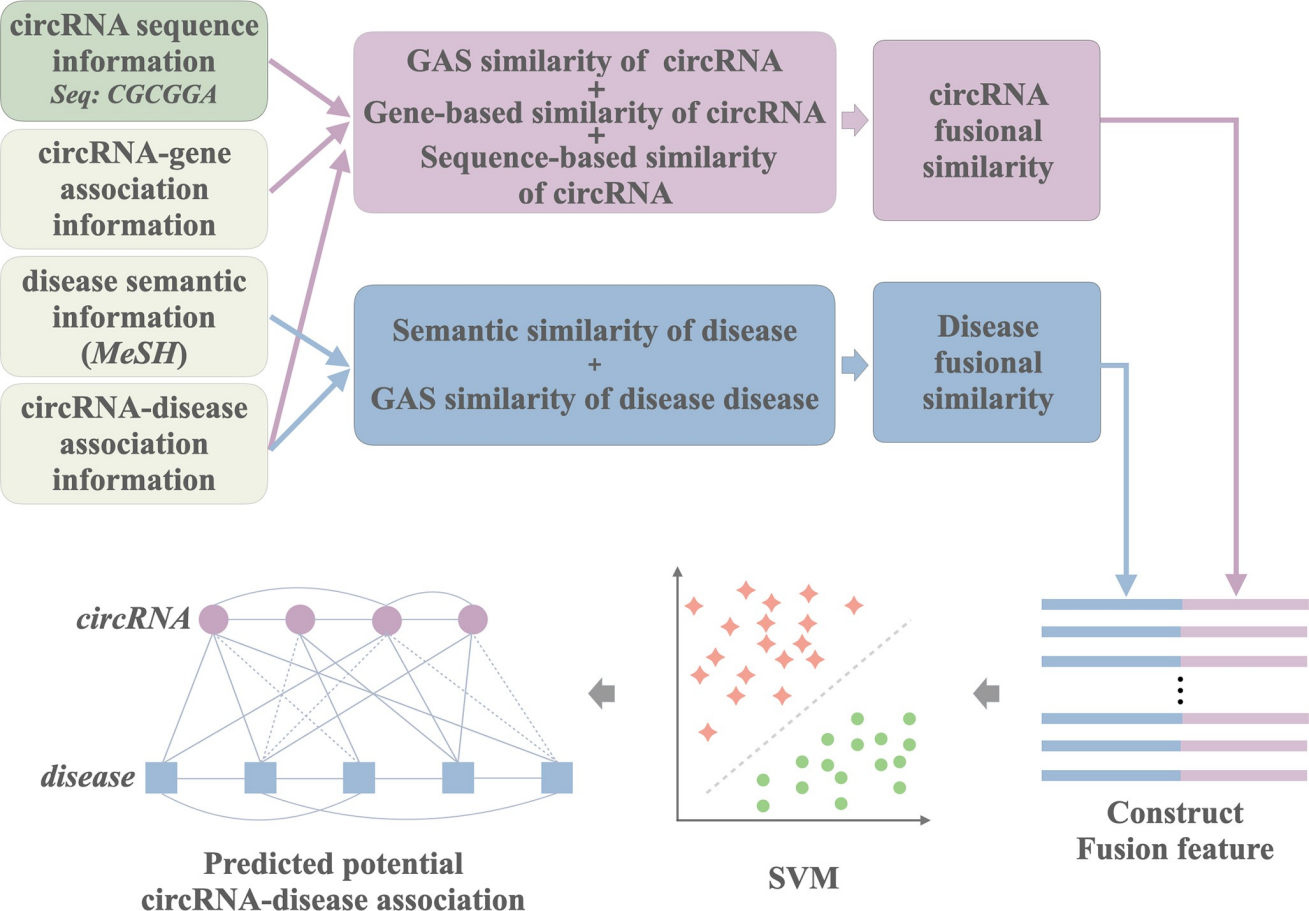
The workflow of iCDA-CGR model to predict potential circRNA-disease associations.

Through the above analysis, we can see that although the current computing models have achieved good results, they also have some defects. First, it is not difficult to see that the training data used by the current model is limited, which has an impact on the robustness of the model. At the same time, the lack of training data also brings the problem of limited coverage. The potential associations that these models can predict are all around 10,000. Secondly, they are mainly based on a single data description method, which does not integrate circRNA and disease behavior information and attribute information in the network to comprehensively define the feature of circRNA and disease, resulting in limited prediction performance. Finally, they did not take the circRNA sequence information into account and cannot accurately measure the circRNA similarity. Therefore, in order to improve the drawbacks of the current computational models, we propose iCDA-CGR model to identify CircRNA-Disease Associations based on Chaos Game Representation. By introducing the circFunBase database and sequence information, the problems of limited model coverage and limited predictive performance are solved. The iCDA-CGR integrates multi-source information, including circRNA sequence information, gene-circRNA associations information, circRNA-disease associations information and the disease semantic information. In particular, iCDA-CGR extracts the biological sequence position information and quantifies the biological sequence nonlinear relationship of circRNA by Chaos Game Representation (CGR) technology [29]. Specifically, iCDA-CGR first figures the disease semantic similarity and disease Gaussian interaction profile kernel (GAS) kernel similarity and combines them to construct disease fusional similarity. Secondly, the method quantizes position and nonlinear sequence information through Chaos Game Representation (CGR) technology to calculate the similarity and difference of circRNAs by Pearson correlation coefficient. Thirdly, circRNA sequence-based similarity, circRNA gene-based similarity and circRNA GAS similarity are integrated into circRNA fusional similarity. Fourthly, feature descriptors are formed by circRNA fusional similarity and disease fusional similarity. Finally, the iCDA-CGR put feature descriptors into support vector machines to predict potential circRNA-disease association. The workflow of iCDA-CGR is shown as Fig 1. We verify the reliability of the method with the five-fold cross-validation on the CircR2Disease database. The average prediction area under curve (AUC) of our method is of 85.14% and the prediction accuracy is 81.12%. Our source code and data can be downloaded on GitHub (https://github.com/look0012/iCDA-CGR). It contains the datasets, the algorithm code and the models. It is worth mentioning that in order to make it more convenient for readers, we provide an easy-to-use version. The user only needs to enter the predicted circRNA and disease name in the following code to perform the prediction operation. The list of circRNAs and diseases is also in the published document, and users can use the list to find the associations they need. There are two models in this version, trained on circR2Disease and CircFunBase respectively. Among them, iCDA-CGR (circR2Disease) can predict 46,825 unconfirmed associations. iCDA-CGR (CircFunBase) can provide predictive scores for approximately 170,000 unconfirmed associations. We hope that these improvements will better serve circRNA researchers as a way to advance the field.

## Methods

### Data sets

#### Benchmark database of circRNA-disease associations

In the past year, a number of benchmark databases have been proposed for collecting circRNA-disease associations, such as circR2Disease, circRNADisease, circFunBase, and Circ2Disease, which contain the association between experimentally validated diseases and circRNAs [22–24]. In this article, circR2Disease and circFunBase are used as the benchmark data set. The detailed description is as follows:

circR2Disease. To evaluate the reliability of our method, the widely used benchmark set circR2Disease was selected. The dataset was preprocessed due to its repetitiveness and non-human circRNA disease association. Specifically, we obtained 612 confirmed circRNA-disease associations consisting of 533 circRNA and 89 diseases after removing the circRNAs in which the gene symbol could not be found, as shown in Table 1. The base dataset circR2Disease can be defined as:

$$Z_{1}=Z_{1}{}^{p}\cup Z_{1}{}^{n}$$ (1)

where *Z*_1_^*p*^ is a positive subset constructed by 612 confirmed circRNA-disease associations, *Z*_1_^*n*^ is a negative subset containing 612 associations which are selected from all 47437 unconfirmed associations between diseases and circRNAs. ∪ is the union of set theory. Known circRNA-disease associations and their names obtained from circR2Disease database can be seen in S1–S3 Tables.

**Table 1:** Data distribution of the benchmark set circR2Disease and circFunBase of circRNA-disease association.

benchmark set	circRNA	Disease	Association
circR2Disease	533	89	612
circFunBase	2597	67	2984

circFunBase. CircFunBase is a database that provides high-quality functional circRNA resources and few models are used. In order to improve the problem of small coverage predicted by the current model, we also performed experiments on this dataset. After removing circRNAs that did not match the gene symbols, 2984 confirmed circRNA-disease associations were obtained, including 2597 circRNAs and 67 diseases, as shown in Table 1. The Benchmark database circFunBase can be defined as:

$$Z_{2}=Z_{2}{}^{p}\cup Z_{2}{}^{n}$$ (2)

where *Z*_2_^*p*^ is a positive subset constructed by 2984 confirmed circRNA-disease associations, *Z*_2_^*n*^ is a negative subset containing 2984 associations which are selected from all 168031 unconfirmed associations between diseases and circRNAs.

#### CircRNAs and their sequence information

Sequence information and gene symbols information for circRNAs are provided by many public databases such as circBase, CIRCpedia, deepBase, CircNet and circRNADb[17–21]. To be able to construct a more complete circRNA sequence dataset, we downloaded circRNA sequence information from a database, circBase. The database is accessible free of charge via the web server http://www.circbase.org/.

### Related work

#### Chaos Game Representation (CGR)

It is an iterative mapping technique for processing sequences[29]. The first advantage of this algorithms is that the original sequence information can be completely recovered from the coordinates. It means that information is not lost in mapping. Secondly, each sequence has a unique mapping, which means that positional information is preserved. For these reasons, the CGR is suitable for transformation of nucleotide sequence. The position *P*_*i*_ was figured by:

$$P_{i}=\nu *(P_{i-1}-g_{i})+P_{i-1}\;i=1\ldots \;n_{seq}$$ (3)

Where *ν* is the nucleotide contribution factor and we set it to be 0.5. *g*_*i*_ is the nucleotide position factor. A, C, G, T are corresponding to (0,0), (0,1), (1,1), (1,0) respectively. *n*_*seq*_ is the length of the sequence and *P*_0_ = (0.5,0.5).

### Similarity between diseases

#### Disease semantic similarity

The Medical Subject Headings (MeSH) database categorizes the disease rigorously, which helps to calculate the semantic similarity of the disease. It can be download from https://www.nlm.nih.gov/ [30]. We can express a disease as a directed acyclic graph (DAG) based on semantic information from the MeSH database. The nodes in DAG represent the diseases, and the edges represent their relationships. If the disease is pathologically similar, more parts of DAG will be shared. Wang et al. [31] proposed a method that has been widely used to calculate the semantic similarity of diseases in recent years. We defined a model for calculating disease contribution values, which is as follows:

$$S_{d(i)}(r)=log(1+\frac{n(DAGs(r))}{n(disease)})$$ (4)

We define the amount of DAGs which includes disease *r* as *n*(*DAGs*(*r*)) and the quantity of all diseases as *n*(*disease*). Therefore, the semantic similarity score $S_{sem}^{D}$ of the disease *d*(*i*) and the disease *d*(*j*) is described as follows:

$$S_{sem}^{D}(d(i),d(j))=\frac{\sum_{r\in N_{d(i)}\cap N_{d(j)}}{(S}_{d(i)}(r)+S_{d(j)}(r))}{\sum_{r\in N_{d(i)}}S_{d(i)}(r)+\sum_{r\in N_{d(j)}}S_{d(j)}(r)}$$ (5)

where *N*_*d*(*i*)_ is defined as all diseases that appear in the disease *d*(*i*)’s DAG.

#### Disease GAS similarity

Many researches have applied Gaussian interaction profile kernel (GAS) to measure the similarity between diseases, according to that pathologically similar diseases tend to be associated with functionally similar circRNAs. In this study, the $S_{GAS}^{D}$ was used to describe the disease similarity information as follow:

$$S_{GAS}^{D}(d(i),d(j))=exp(-\tau_{d}{\left||A_{cd}(d(i))-A_{cd}(d(j))|\right|}^{2})$$ (6)

Where

$$\tau_{d}=\frac{1}{\frac{1}{m}\sum_{i=1}^{m}{\left||A_{cd}(d(i))|\right|}^{2}}$$ (7)

$$A_{cd}=\left[\begin{array}{ccc} t_{1,1} & \cdots & t_{1,m}\\ \vdots & \ddots & \vdots \\ t_{n,1} & \cdots & t_{n,m} \end{array}\right]$$ (8)

We define the parameter as the width parameter of the function, *τ*_*d*_. The quantity of diseases and circRNAs are defined as *m* and *n* represently. Association adjacency matrix *A*_*cd*_ represents the positive subset *Z*_*p*_. If circRNA *r*(*i*) and disease *r*(*j*) have an association, element *t*_*i*,*j*_ is set to be 1, otherwise 0. *A*_*cd*_(*d*(*i*)) is association profiles of disease *d*(*i*). Here, we utilize the *i*th column vector of the adjacency matrix to describe *A*_*cd*_(*d*(*i*)).

#### Disease fusional similarity

By analyzing the disease similarity measures form multiple perspectives, we gain the similarity matrices, including $S_{sem}^{D}$ and $S_{GAS}^{D}$. However, some of semantic similarity are unable to be calculated if the disease does not have its own DAG. To compensate for this deficiency, we will fuse $S_{sem}^{D}$ and $S_{GAS}^{D}$ like the previous researches [32–34]. The disease fusional similarity *S*^*D*^ between disease *d*(*i*) and *d*(*j*) is defined as follow, and the final disease similarity matrix can be seen in S4 Table.

$$S^{D}(d(i),d(j))=\left\{ \begin{array}{l} \frac{S_{sem}^{D}(d(i),d(j))+S_{GAS}^{D}(d(i),d(j))}{2}\;if\;d(i)and\;d(j)have\;DAG\\ \;S_{GAS}^{D}(d(i),d(j))\;otherwise \end{array}\right.$$ (9)

### Similarity between circRNAs

#### CircRNA gene-based similarity

Circular RNA regulates the activity of RNA polymerase and promotes parental genes’ transcription found in previous researches. Because if RNA affects the same human disease, their functions tend to be similar [35–37]. In this work, we downloaded gene-circRNA association information from crcR2Disease database. The circRNA gene-based similarity matrix was constructed as follow:

$$S_{gene}^{C}=A_{cg}\times S_{gas}^{G}\times {A_{cg}}^{T}$$ (10)

Where the elements in $S_{gene}^{C}$ is functional similarity scores between circRNAs. Association adjacency matrix *A*_*cg*_ represents the association between genes and circRNA. If gene target and circRNA have an association, the element of *A*_*cg*_ is set to be 1, otherwise 0. The gene’s GAS similarity matrix $S_{gas}^{G}$ is constructed by Association adjacency matrix *A*_*cg*_. *T* is the transpose operator.

#### CircRNA GAS similarity

Many researches chose to utilize gaussian interaction profile kernel (GAS) to measure the similarity between biomolecules [38]. Because if RNA affects the same human disease, their functions tend to be similar [35–37]. In this study, the $S_{GAS}^{C}$ was used to describe the circRNA similarity information as follow:

$$S_{GAS}^{C}(c(i),c(j))=exp(-\tau_{c}{\left||A_{cd}(c(i))-A_{cd}(c(j))|\right|}^{2})$$ (11)

$$\frac{1}{\tau_{c}=\frac{1}{n}\sum_{i=1}^{n}{\left||A_{cd}(c(i))|\right|}^{2}}$$ (12)

Where $S_{GAS}^{C}(c(i),c(j))$ is the GAS similarity value between circRNAs *c*(*i*) and circRNAs *c*(*j*). The *i* -th row vector in the adjacency matrix *A*_*cd*_ is defined as the association profile *A*_*cd*_(*c*(*i*)) of circRNA *c*(*i*), which is a vector composed of the relationship between circRNA *c*(*i*) and all diseases. *τ*_*c*_ is the width parameter.

#### circRNA sequence-based similarity

Existing sequence alignment algorithms only quantify position information or non-linear information, and few algorithms that can combine both are proposed. Therefore, a new CGR-based method is proposed to quantify the similarity and difference between position and non-linear information using Pearson correlation coefficient. The specific calculation process is as follows.

**Fig 2:**
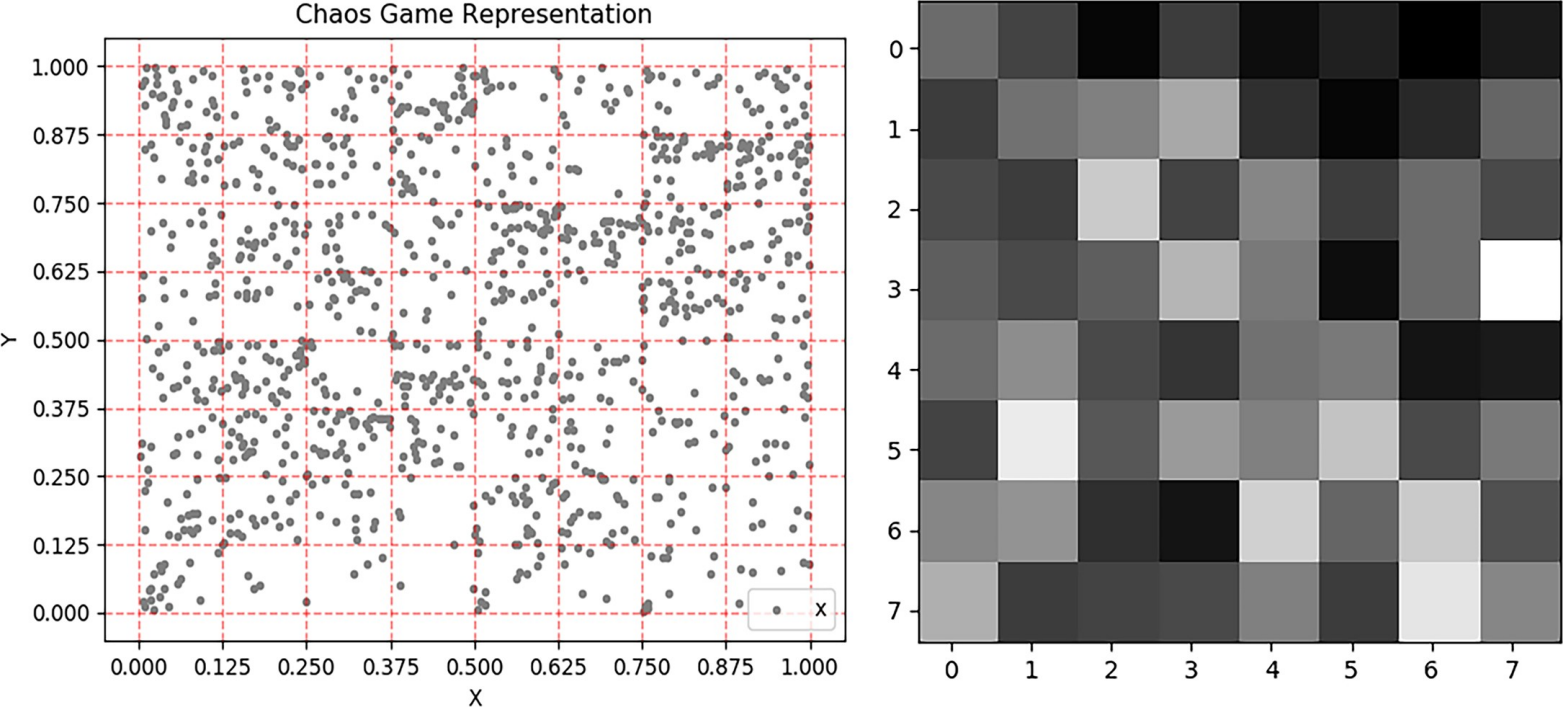
A) the CGR of hsa_circ_0005931 are plotted with the average coordinates for each 8 × 8 quadrant represented. B) A matrix of hsa_circ_0005931’s nucleotides with probabilities for chaos game representation.

Firstly, the CGR space is divided into *N*_*g*_ *grid* (*N*_*g*_ = 2^*s*^×2^*s*^,s = 3), as Fig 2. And, *grid* can be represented as formula 13.

$${grid}_{i}=(X_{i},Y_{i},Z_{i})$$ (13)

Secondly, the abscissa *point*.*x* and ordinate *point*.*y* in each grid are accumulated respectively to quantify position information.

$$X_{i}=\sum point.x\;if\;points\;in\;{grid}_{i}$$ (14)

$$Y_{i}=\sum point.y\;if\;points\;in\;{grid}_{i}$$ (15)

Thirdly, we calculate the z-scores of each grid *Z*_*i*_ to quantify nonlinear information.

$$Z_{i}=\frac{{Num}_{i}-\frac{\sum_{k=1}^{N_{g}}{Num}_{k}}{N_{g}}}{\sqrt{\frac{1}{N_{g}}\sum_{h=1}^{N_{g}}{\left({Num}_{h}-\frac{\sum_{f=1}^{N_{g}}{Num}_{f}}{N_{g}}\right)}^{2}}}$$ (16)

$${Num}_{i}=number\;of\;points\;in\;{grid}_{i}$$ (17)

**Fig 3:**
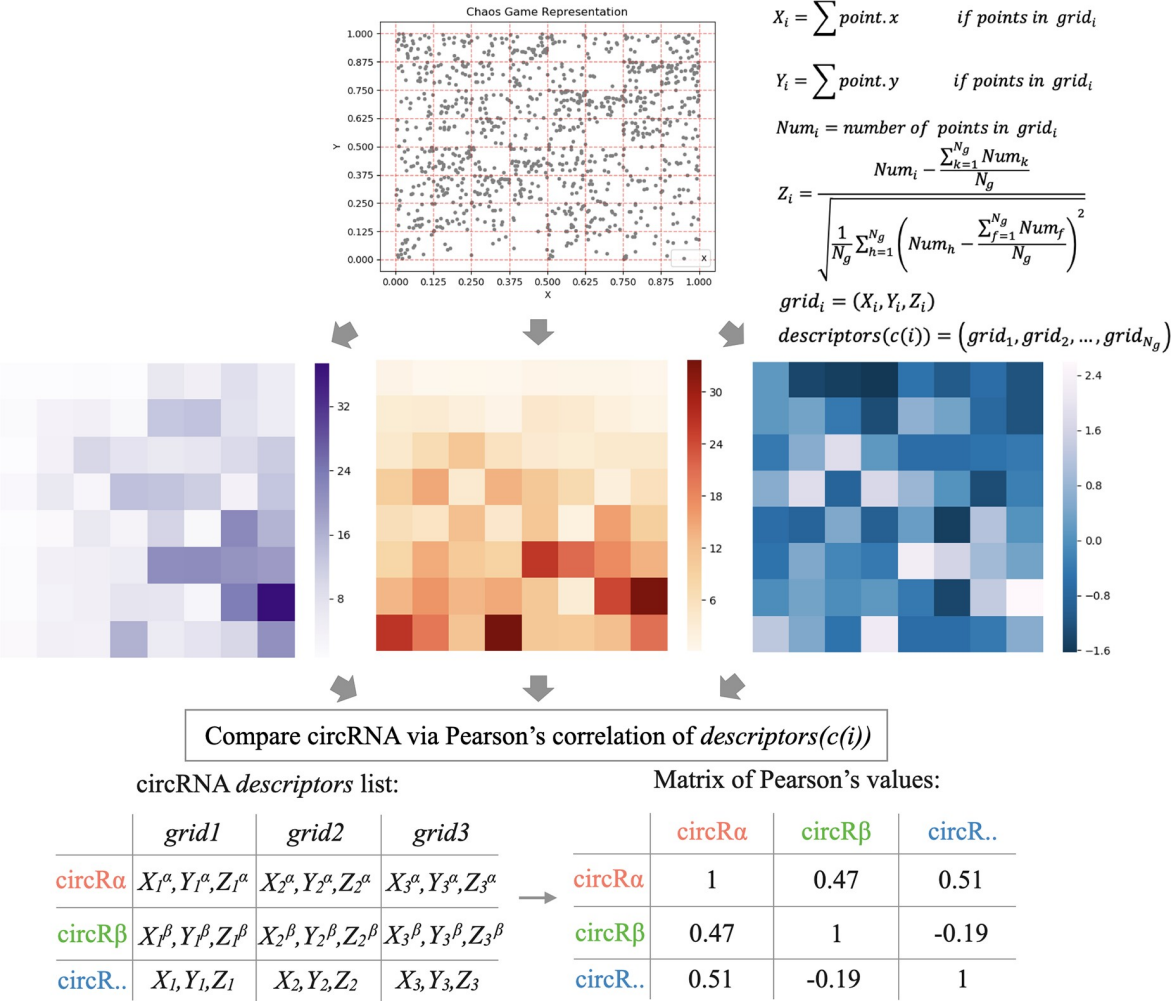
The workflow of circRNA sequence-based similarity.

Finally, each grid can be described as three attributes, and we fused the attributes to construct the descriptors *descriptors*(*c*(*i*)) to determine the sequence similarity $S_{seq}^{C}(c(i),c(j))$ by Pearson correlation coefficient. Where *c*(*i*) represents the *i* -th cricRNA. The workflow is shown as Fig 3.

$$S_{seq}^{C}(c(i),c(j))=\frac{Cov\left(descriptors(c(i)),descriptors(c(j)\right))}{D(descriptors(c(i)))*D(descriptors(c(j)))}$$ (18)

$$descriptors(c(i))=({grid}_{1},{grid}_{2},\ldots ,{grid}_{N_{g}})$$ (19)

where *Cov*(*descriptors*(*c*(*i*))) is the covariance of *descriptors*(*c*(*i*)), *D*(*descriptors*(*c*(*i*))) is the variance of *descriptors*(*c*(*i*)). The size of circRNA sequence similarity matrix $S_{seq}^{C}(c(i),c(j))$ is *n*×*n*. All sequence information used in this article was downloaded from circBase [17].

#### CircRNA fusional similarity

By analyzing circRNA’s characteristics from different perspectives, we can obtain three similarity matrices, including $S_{gene}^{C}$ (formula 8), $S_{GAS}^{C}$ (formula 9), and $S_{seq}^{C}$ (formula 16). Since the two adjacency matrices *A*_*cd*_ and *A*_*cg*_ are sparse, the two similarities $S_{gene}^{C}$ and $S_{GAS}^{C}$ obtained by collaborative filtering have no significant difference in value and can't effectively distinguish circRNA. In order to solve the small difference between circRNAs due to lack of data and availability, we try to describe circRNA from a different perspective to make it more informative. To this end, the sequence similarity is introduced. However, some circRNAs lack sequence information corresponding to the experiment. So, the completion of similarity information is accomplished by combining three matrices. The fusional similarity *S*^*C*^ is defined as follow, and the final circRNA similarity matrix can be seen in S5 Table.

$$S^{C}=\left\{ \begin{array}{l} \frac{S_{gene}^{C}(c(i),c(j))+S_{GAS}^{C}(c(i),c(j))+S_{seq}^{C}(c(i),c(j))}{3}\;if\;S_{seq}^{C}(c(i),c(j))\neq 0\\ \;\frac{S_{gene}^{C}(c(i),c(j))+S_{GAS}^{C}(c(i),c(j))}{2}\;otherwise \end{array}\right.$$ (20)

### Prediction of association between circRNA and disease by SVM

Support Vector Machines (SVM) was introduced in 1963 by Vanpik *et al*., which demonstrated many unique advantages in solving small sample, nonlinear and high dimensional pattern recognition problems. Due to the training samples used in iCDA-CGR are small, SVM is selected to build a model of predicting potential circRNA-disease association. Prediction is mainly divided into three steps: 1. Construct positive and negative sample sets; 2. Form the association descriptors based on the characteristics of the circRNA and disease; 3. Train models based on descriptors to predict potential circRNA-disease associations. Each step will be described in detail below.

Firstly, we built positive and negative sample sets. Specifically, 612 corresponding experimentally supported circRNA-disease pairs in circR2Disease were chosen as positive samples. Meantime, we randomly selected the same number of associations that without experimentally supported as negative samples.

Secondly, the association descriptors based on the characteristics of the circRNA and disease were formed. We calculated the semantic similarity $S_{sem}^{D}$ and the GAS similarity $S_{GAS}^{D}$ of the disease separately, and integrated them into a matrix *S*^*D*^, and used the similarity of the disease *d*(*i*_*d*_) with all diseases including itself (the *i*_*d*_th row of the matrix *S*^*D*^) as the characteristic descriptor of the disease defined as follow:

$$S^{D}(d(i_{d}))=(v_{1},v_{2},v_{3},\ldots ,v_{m})$$ (21)

where *S*^*D*^(*d*(*i*_*d*_)) represents the *i*th row of the matrix *S*^*D*^. *v*_1_ is the similarity value of *d*(*i*_*d*_) and *d*(1). The size of *S*^*D*^(*d*(*i*_*d*_)) is 1×*m*. At the same time, we calculated the gene-based similarity $S_{gene}^{C}$, the GAS similarity $S_{GAS}^{C}$ and sequence-based similarity of the circRNA separately to form circRNA fusional similarity *S*^*C*^. Using the similarity of the circRNA *c*(*i*_*c*_) with all circRNA including itself (the *i*th row of the matrix *S*^*C*^) describes the characteristic descriptor of the circRNA defined as follow:

$$S^{C}(c(i_{c}))=(w_{1},w_{2},w_{3},\ldots ,w_{n})$$ (22)

where *S*^*C*^(*c*(*i*_*c*_)) represents the *i*th row of the matrix *S*^*C*^. The similarity value between *c*(*i*_*c*_) and *c*(1) is defined as *w*_1_. The size of *S*^*C*^(*c*(*i*_*c*_)) is 1×*n*. circRNA disease samples can be defined as 622-dimensional association descriptors combined *S*^*D*^(*d*(*i*)) and *S*^*C*^(*c*(*i*_*c*_)):

$$F=\left(S^{D}(d(i_{d})),S^{C}(c(i_{c}))\right)=(f_{1},f_{2},f_{3},\ldots ,f_{n+m})$$ (23)

where (*f*_1_,*f*_2_,*f*_3_,…,*f*_*m*_) is *i*_*d*_th row of the disease fusional similarity *S*^*D*^, the *i*_*c*_th row of the circRNA fusional similarity *S*^*C*^ is defined as (*f*_*m*+1_,*f*_*m*+2_,*f*_*m*+3_,…,*f*_*m*+*n*_).

Finally, support vector machines (SVM) is utilized to train samples to build predictive models. More specifically. Firstly, we set the label of the training set. If the samples are in *Z*_*p*_, the label is defined as 1. Meanwhile, if the samples are in *Z*_*n*_, the label is defined as 0. Secondly, we fed the training data into support vector machines (SVM) to get prediction model. By predicting, the higher the score of the circRNA-disease association, the more likely it is the candidate for the potential association.

## Results

### Performance Evaluation

#### The five-fold cross-validation(5-CV)

In this work, the five-fold cross-validation (5-CV) is selected to evaluate the effectiveness of iCDA-CGR in predicting disease-related circRNAs. We separated the base dataset *Z* into five parts on average:

$$\left\{ \begin{array}{c} Z=Z_{1}\cup Z_{2}\cup Z_{3}\cup Z_{4}\cup Z_{5}\\ \emptyset =Z_{1}\cap Z_{2}\cap Z_{3}\cap Z_{4}\cap Z_{5} \end{array}\right.$$ (24)

where ∅ is empty set. ∪ and ∩ are the union and intersection of set theory. Subset *Z*_*i*_, *Z*^*p*^, *Z*^*n*^ can be defined as:

$$\left\{ \begin{array}{l} Z_{i}=Z_{i}^{p}\cup Z_{i}^{n}\\ Z^{p}=Z_{1}^{p}{\cup Z}_{2}^{p}\cup Z_{3}^{p}\cup Z_{4}^{p}\cup Z_{5}^{p}\\ Z^{n}=Z_{1}^{n}\cup Z_{2}^{n}\cup Z_{3}^{n}{\cup Z}_{4}^{n}{\cup Z}_{5}^{n} \end{array}\right.\;i=1,2,3,4,5$$ (25)

The relationship between the *i*th positive subset $Z_{i}^{p}$ or the *i*th negative $Z_{i}^{n}$ can be expressed as:

$$\left\{ \begin{array}{l} {num(Z}_{1}^{p})\cup {num(Z}_{2}^{p})\cup {num(Z}_{3}^{p})\cup {num(Z}_{4}^{p})\cup {num(Z}_{5}^{p})\\ {num(Z}_{1}^{n})\cup {num(Z}_{2}^{n})\cup {num(Z}_{3}^{n})\cup {num(Z}_{4}^{n})\cup {num(Z}_{5}^{n}) \end{array}\right.$$ (26)

where the quantity of sample in the *i*th positive subset $Z_{i}^{p}$ are described as ${num(Z}_{i}^{p})$. In same way, we described the quantity of sample in the *i*th negative subset $Z_{i}^{n}$ as ${num(Z}_{i}^{n})$. In the iCDA-CGR, we utilized four of the positive subset and negative $Z_{i}^{n}$ as the training set and the remaining one as the test set as a cross-validation. The cross-validation is repeated 5 times, and each test set is verified once, with an average of 5 results, and finally a final estimate is obtained.

#### Evaluation criteria

Three evaluation criteria were introduced for assessing the performance of iCDA-CGR. *Accu*. is the ratio of the number of samples correctly classified by the classifier to the total number of samples.

$$Accu.=\frac{TP+TN}{TP+TN+FP+FN}$$ (27)

where *TP* and *FP* are the number of true positive and false positive samples, respectively. *TN* and *FN* are the number of true negative and false negative samples, respectively. *Sen*. is the ratio of the number of samples correctly classified by the classifier to the total positive samples.

$$Sen.=\frac{TP}{TP+FN}$$ (28)

*Prec*. is the ratio of the number of samples correctly classified by the classifier to the sum of true positive and false positive samples.

$$Prec.=\frac{TP}{TP+FP}$$ (29)

*F*_1_ is a comprehensive evaluation index of *Sen*. and *Prec*.

$$F_{1}=\frac{Sen.\times Prec.}{Sen.+Prec.}$$ (30)

### Assessment of prediction ability

**Fig 4:**
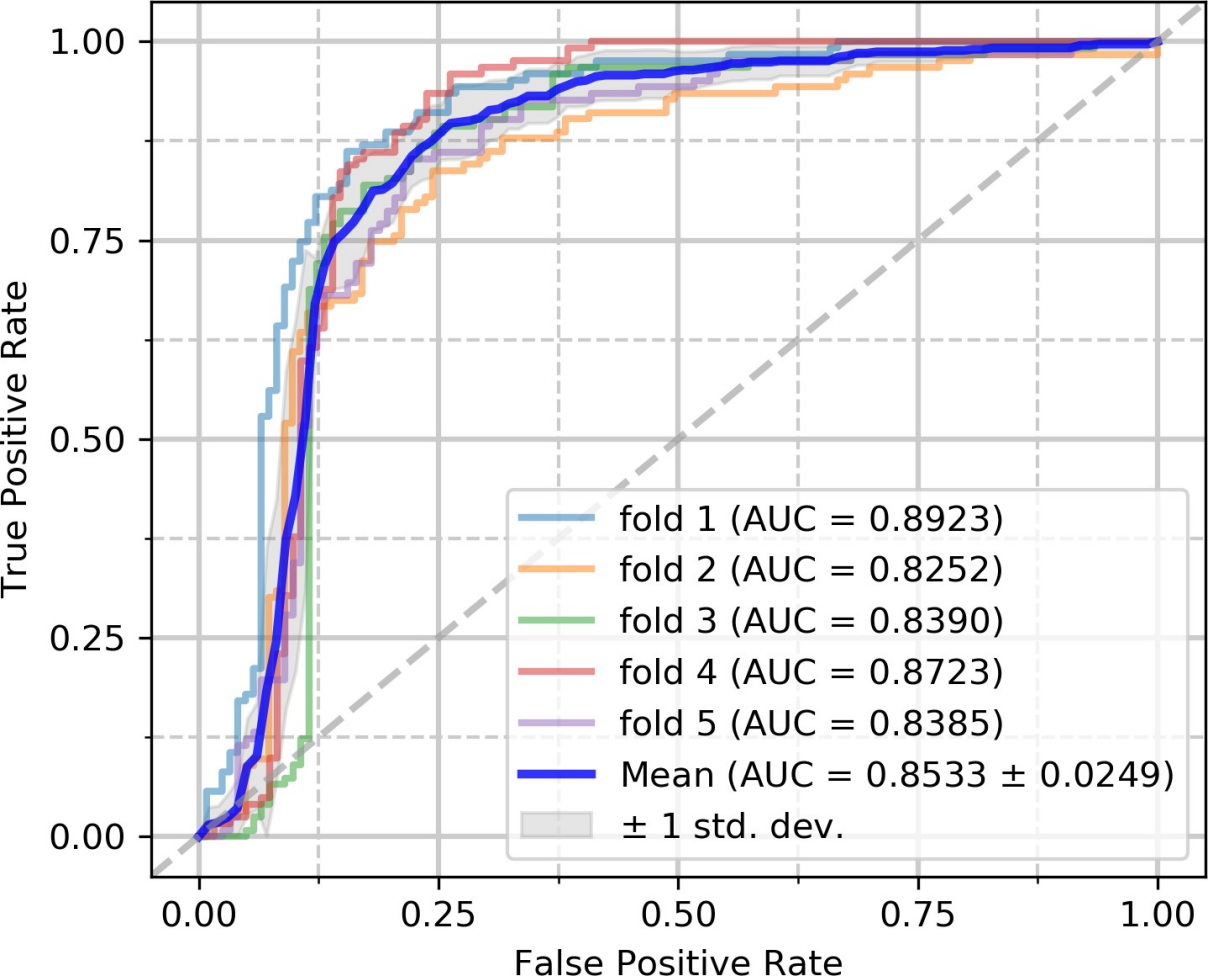
ROC curves performed by iCDA-CGR on circR2Disease dataset.

**Fig 5:**
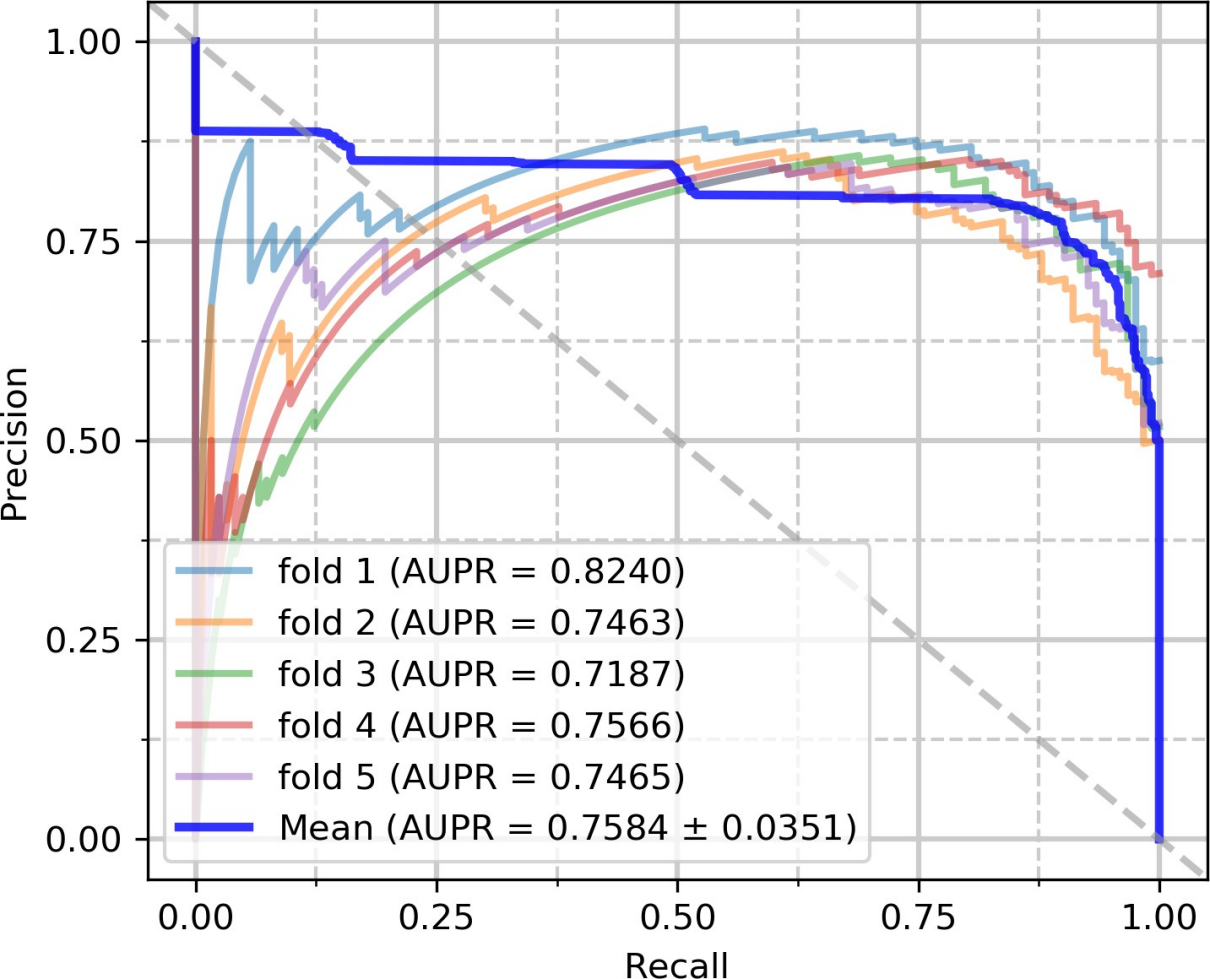
PR curves performed by iCDA-CGR on circR2Disease dataset.

To evaluate the capabilities of the model, we performed experiments on the circR2Disease and circFunBase datasets, respectively. The five-fold cross-validation results on the circR2Disease dataset are summarized in Table 2. iCDA-CGR has gained an average prediction AUC of 0.8533+/-0.0249. The AUCs of the five experiments are 0.8923 (fold 1), 0.8252 (fold 2), 0.8390 (fold 3), 0.8723 (fold 4) and 0.8385 (fold 5) respectively as Fig 4. iCDA-CGR has gained an average prediction AUPR of 0.7584+/-0.0351. The AUPRs of the five experiments are 0.8240 (fold 1), 0.7463 (fold 2), 0.7187 (fold 3), 0.7566 (fold 4) and 0.7465 (fold 5) respectively as Fig 5. The yielded averages of accuracy, sensitivity, precision and f1-score come to be 81.95%, 88.08%, 78.46% and 82.97% as in Table 2.

**Table 2:** The five-fold cross-validation results performed by iCDA-CGR on circR2Disease dataset.

Testing set	Accuracy	Precision	Sensitivity	F1-score
1	83.74%	80.74%	88.62%	84.50%
2	78.86%	76.30%	83.74%	79.84%
3	81.15%	76.76%	89.34%	82.58%
4	84.84%	79.72%	93.44%	86.04%
5	81.15%	78.79%	85.25%	81.89%
Average	81.95±2.11%	78.46±1.70%	88.08±3.39%	82.97±2.14%

**Fig 6:**
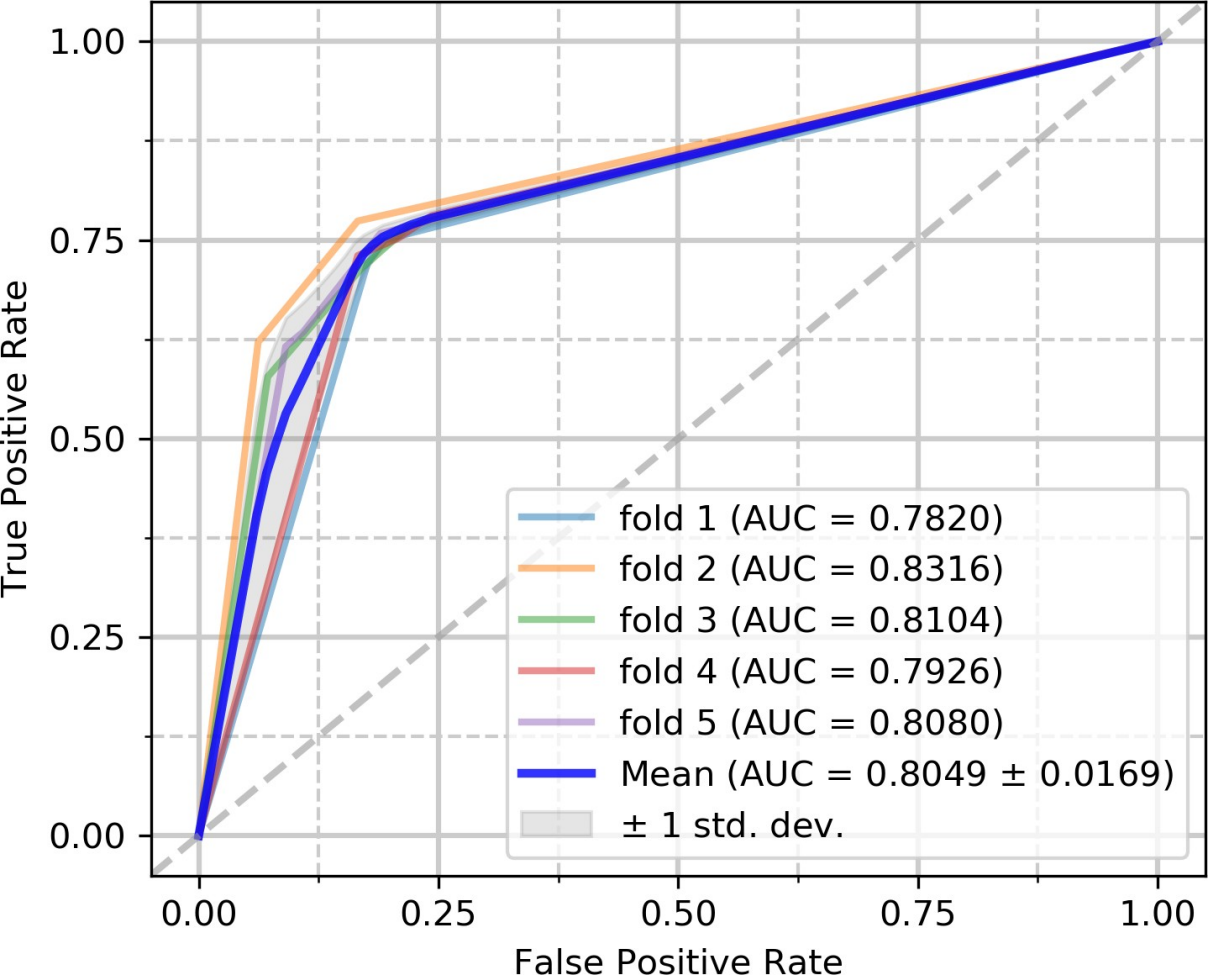
ROC curves performed by iCDA-CGR on circFunBase dataset.

**Fig 7:**
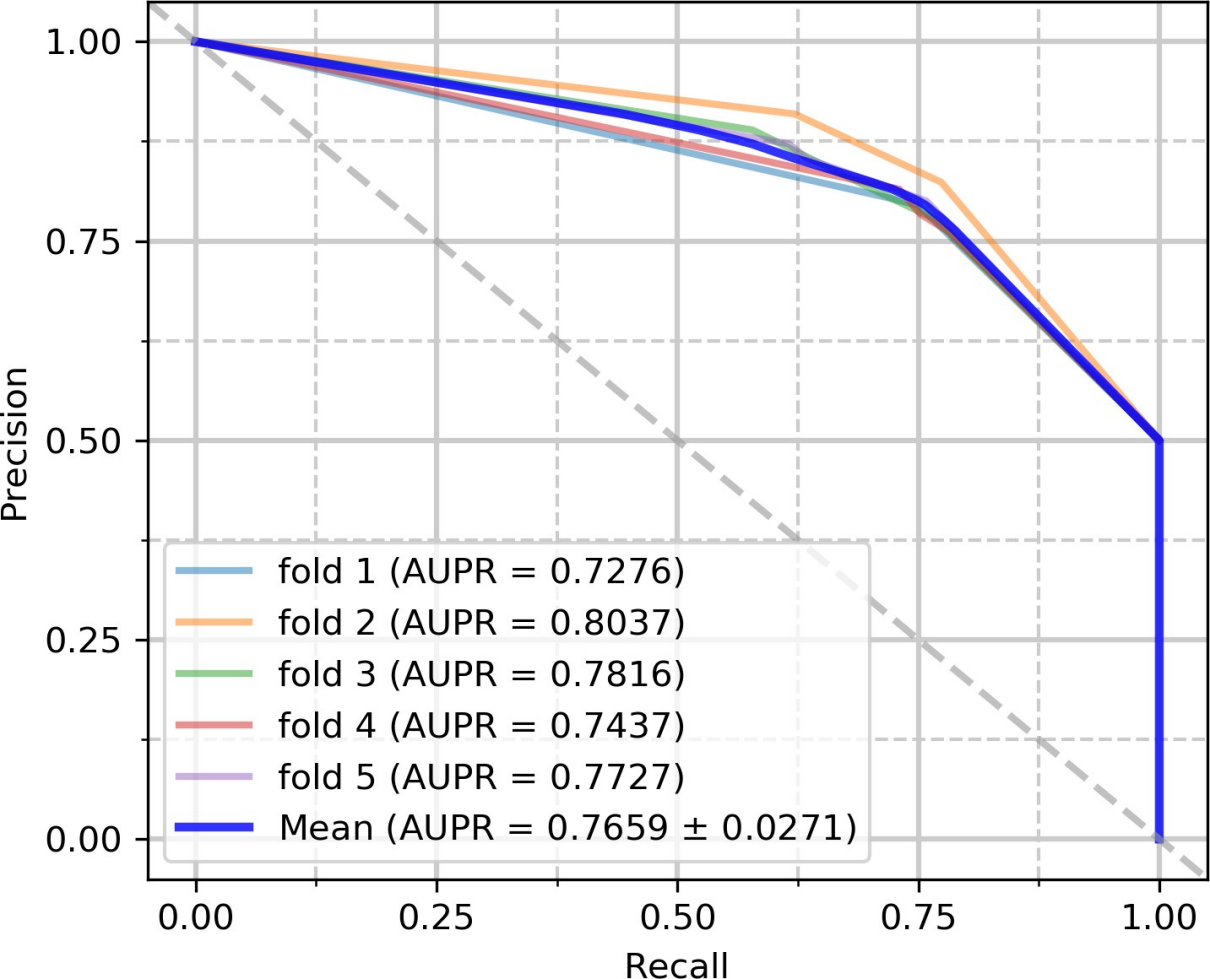
PR curves performed by iCDA-CGR on circFunBase dataset.

On the circFunBase dataset, the mean and standard deviation were utilized as the experimental results of the five-fold cross-validation. In Table 3, the experimental results were obtained by iCDA-CGR on the circFunBase database. iCDA-CGR has gained an average prediction AUC of 0.8049+/-0.169. The AUCs of the five experiments are 0.7820 (fold 1), 0.8316 (fold 2), 0.8104 (fold 3), 0.7926 (fold 4) and 0.8080 (fold 5) respectively as Fig 6. The AUPRs of the five experiments are 0.7276 (fold 1), 0.8037 (fold 2), 0.7816 (fold 3), 0.7437 (fold 4) and 0.7727 (fold 5) respectively as Fig 7. The yielded averages of accuracy, precision, sensitivity and f1-score come to be 78.03%, 79.96%, 74.94% and 77.31% as in Table 3.

**Table 3:** The five-fold cross-validation results performed by iCDA-CGR on circFunBase dataset.

Testing set	Accuracy	Precision	Sensitivity	F1-score
1	77.22%	80.37%	72.03%	75.97%
2	80.40%	82.35%	77.39%	79.79%
3	77.22%	80.83%	71.36%	75.80%
4	76.88%	76.27%	78.06%	77.15%
5	78.44%	80.00%	758.4%	77.86%
Average	78.03±1.30%	79.96±2.01%	74.94±2.75%	77.31±1.45%

### Comparison among different classifiers

In the above experiment, iCDA-CGR has received a reliable result. To prove the correctness of the classifier selection, we have compared the support vector machine (SVM) with random forest (RF), decision tree (DT), k-nearest neighbor (KNN) on benchmark database circR2Disease.

Support vector machines (SVM) is a binary classification model. Its purpose is to find a hyperplane to segment samples. The principle of segmentation is to maximize the spacing, and finally it is transformed into a convex quadratic programming problem to solve. The decision tree (DT) adopts a top-down recursive method. The basic idea is to construct a tree with the fastest entropy decline as measured by information entropy, and the entropy value at the leaf node is 0. The random forest (RF) is a kind of Ensemble Learning, which belongs to Bagging. By combining multiple weak classifiers, the final results can be voted or averaged, which makes the results of the whole model have higher accuracy and generalization performance. The main idea of the k-nearest neighbor (KNN) algorithm is that if most of the k most adjacent samples in the feature space belong to a certain category, then the sample also belongs to this category and has the characteristics of samples in this category.

**Fig 8:**
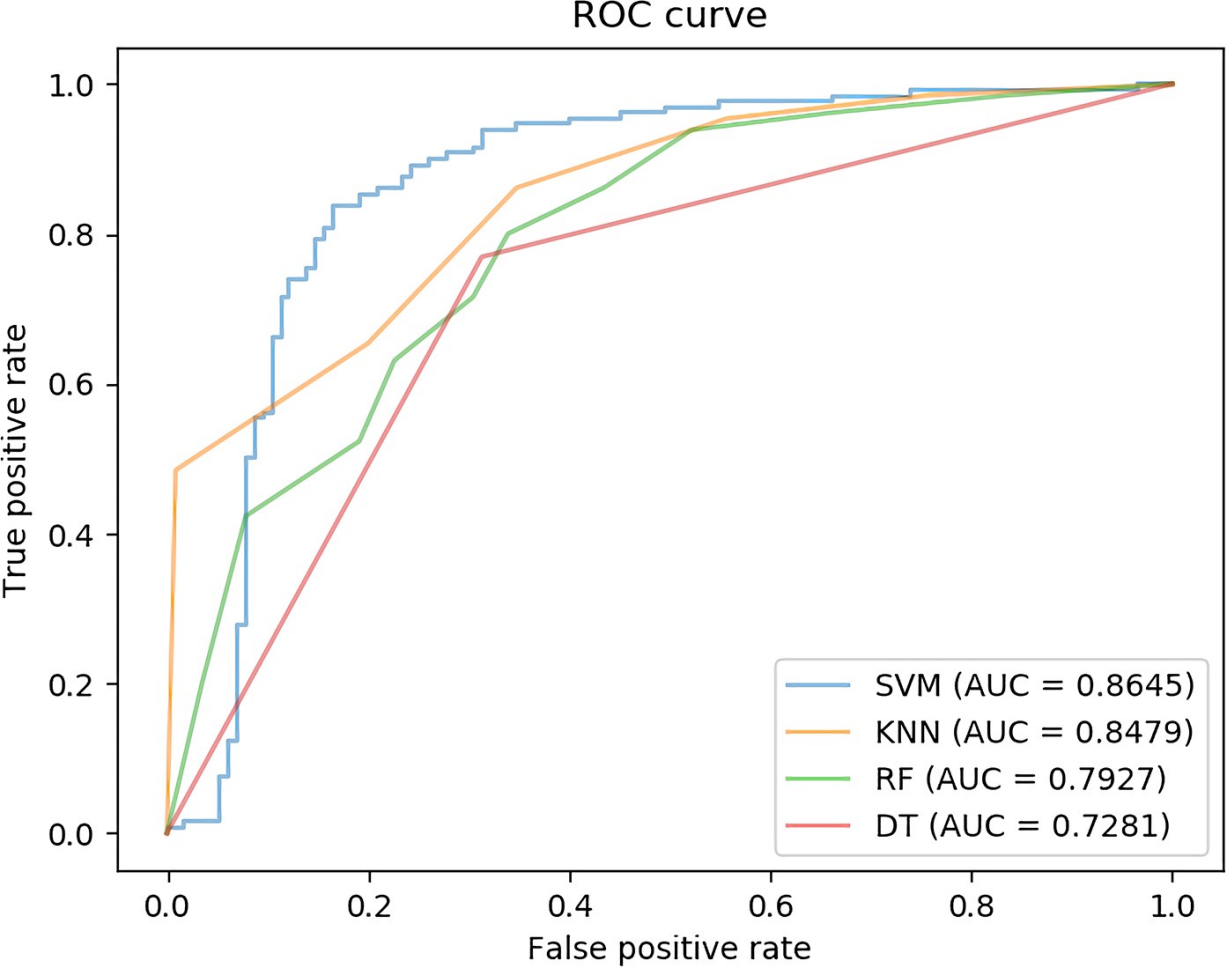
The ROCs of four different classifiers which are support vector machines, decision tree, random forest and k-nearest neighbor on circR2Disease dataset.

In Table 4, we compare the results of Support vector machines with the other three classifiers on the circR2Diseas database. The accuracy of the four experiments are 82.44% (Support vector machines), 76.32% (k-nearest neighbor), 70.61% (Random forest) and 73.06% (Decision Tree). Their AUC are 0.8645 (Support vector machines), 0.8479 (k-nearest neighbor), 0.7927 (Random forest) and 0.7281 (Decision Tree) shown as Fig 8.

**Table 4:** Performance comparison among four different classifiers which are k-nearest neighbor, random forest, decision tree and support vector machine.

Method	Accuracy (%)	Sensitivity (%)	Precision (%)	F1-score (%)
KNN	76.32%	86.15%	73.68%	79.43%
RF	70.61%	71.54%	72.66%	72.09%
DT	73.06%	76.39%	73.53%	75.19%
**SVM**	**82.44%**	**87.69%**	**80.85%**	**84.13%**

### Comparison with related models

To further evaluate the reliability of iCDA-CGR, we compared it to five related prediction models: KATZHCDA, GHICD, RWRHCD, CD-LNLP and ICFCDA. The details of the comparison are summarized in Table 5. From the table, we can see that KATZHCDA, GHICD, RWRHCD and our model iCDA-CGR are all based on circR2Disease data set and use the five-fold cross-validation method, so iCDA-CGR can be directly compared with these three models. In terms of AUC scores reflecting the overall performance of the model, KATZHCDA, GHICD and RWRHCD achieved 0.7936, 0.7290 and 0.6660 respectively, while the proposed model iCDA-CGR achieved 0.8533. The results show that iCDA-CGR is significantly better than these methods.

**Table 5:** Performance comparison (AUC scores) among four different prediction model which are iCDA-CGR, KATZHCDA, GHICD, RWRHCD and CD-LNLP, ICFCDA.

Method	AUC	Dataset	Association	Assessment method
GHICD	0.7290	circR2Disease	592	5-CV^a^
KATZHCDA	0.7936	circR2Disease	312	5-CV^a^
RWRHCD	0.6660	circR2Disease	592	5-CV^a^
**iCDA-CGR**	**0.8533**	**circR2Disease**	**612**	**5-CV**^a^
CD-LNLP	0.9007	circ2Disease	273	LOOCV^b^
ICFCDA	0.9460	circR2Disease	212	LOOCV^b^

^**a**^ 5-CV is short for five-fold cross-validation

^**b**^ LOOCV is short for leave-one-out cross-validation

In the last two rows of Table 5, we list the performance of CD-LNLP and ICFCDA, which are 0.9007 and 0.9460, respectively. However, because the dataset or assessment methods used by these two models are inconsistent with the proposed model, we cannot directly compare them, so they are used as a reference for model performance. The specific reasons that cannot be directly compared are as follows:

For model CD-LNLP, it uses the circ2Disease database instead of the more commonly used circR2Disease database. Due to the different data sources used, the training model evaluation criteria will be different. Furthermore, CD-LNLP uses leave-one-out cross validation (LOOCV) to evaluate model performance instead of the more commonly used five-fold cross validation (5-CV). Based on previous work, using the same model and data, LOOCV assessments are usually higher than 5-CV [39]. Therefore, CD-LNLP cannot be directly compared with the proposed model.

For model ICFCDA, it uses the circR2Disease database, but this method removes more noisy data. The training data of ICFCDA includes 212 associations consisting of 200 circRNAs and 42 diseases. The predicted coverage of this model is 7976 associations, which is 17.25% of the coverage of iCDA-CGR. This operation makes the model performance stronger, but sacrifices the model's coverage. In addition, ICFCDA also uses LOOCV. Therefore, ICFCDA cannot be directly compared with the proposed model.

In summary, the proposed model has superior performance and coverage, which indicates that CGR-based sequence extraction technology and characterization of intrinsic structure and circRNA-disease association information could effectively improve the reliability of prediction.

### Case study

To verify the performance of the model in predicting potential associations based on confirmed associations, we carried out a case study. To be specific, we define the training samples and test samples as follows:

$$\left\{ \begin{array}{c} {Z_{1}}^{train}=Z_{1}\\ {Z_{1}}^{test}=C_{U}Z_{1} \end{array}\right.$$ (31)

In the validation, confirmed associations *Z*_1_ between circRNA and disease provided by the circR2Disease database were selected as training set *Z*_1_^*train*^. Meanwhile, all the possible association are selected as test sets *Z*_1_^*test*^. The size of *Z*_1_^*train*^ and *Z*_1_^*test*^ are 1224 and 46213 respectively. Here, we verified the top 30 associations with the highest score. Among them, 19 pairs were verified in different literatures shown as Table 6.

$$\left\{ \begin{array}{c} {Z_{2}}^{train}=Z_{2}\\ {Z_{2}}^{test}=C_{U}Z_{2} \end{array}\right.$$ (32)

**Table 6:** Prediction of the top 30 predicted circRNAs associated based on known associations on circR2Disease.

Rank	circRNA	Disease	Evidence (PMID)
1	Circ_MED12L	Hepatoblastoma	unconfirmed
2	hsa_circ_0070933	Oral squamous cell carcinoma	unconfirmed
3	hsa_circ_0070934	Diabetic myocardial fibrosis	unconfirmed
4	hsa_circ_0002113	Breast cancer	28803498
5	hsa_circ_0070934	Hypertension	unconfirmed
6	hsa_circ_0067934	Hepatocellular carcinoma	29458020
7	hsa_circ_0001445	Pancreatic cancer	unconfirmed
8	hsa_circ_0014717	Gastric cancer	28544609
9	hsa_circ_0001649	Gastric cancer	28167847
10	hsa_circ_0001649	Glioma	29343848
11	hsa_circ_0067934	Esophageal squamous cell carcinoma	27752108
12	hsa_circ_0003838	Breast cancer	28803498
13	circETFA	Breast cancer	29221160
14	mmu_circ_0001052	Immunosenescence	unconfirmed
15	circMED13	Breast cancer	29221160
16	hsa_circ_0068087	Rheumatoid arthritis	unconfirmed
17	hsa_circ_0007031	Colorectal cancer	28656150
18	hsa_circ_0068033	Breast cancer	29045858
19	Circ_SMARCA5	Glioma	26873924
20	hsa_circ_0000504	Colorectal cancer	28656150
21	circ-Foxo3	Acute ischemic stroke	unconfirmed
22	hsa_circ_0072359	Hepatoblastoma	29414822
23	Circ_ZNF148	Glioma	26873924
24	hsa_circ_0081342	Papillary thyroid carcinoma	28288173
25	mmu_circ_0000290	Primary great saphenous vein varicosities	unconfirmed
26	circ-FBXW7	Glioblastoma	28903484
27	hsa_circ_0085495	Breast cancer	28803498
28	hsa_circ_0001824	Breast cancer	unconfirmed
29	Circ_ADCY1	Glioma	26873924
30	circDLGAP4	Cardiovascular disease	unconfirmed

Similar to the definition above, the confirmed associations provided by the circFunBase database were selected as the training set *Z*_2_^*train*^. At the same time, all possible associations are selected as test set *Z*_2_^*test*^. The size of *Z*_2_^*train*^ and *Z*_2_^*test*^ are 5968 and 168031 respectively. Here, we verified the top 30 correlations with the highest score. And, 17 pairs were verified in different literatures shown as Table 7.

**Table 7:** Prediction of the top 30 predicted circRNAs associated based on known associations on circFunBase.

Rank	circRNA	Disease	Evidence (PMID)
1	hsa_circ_0078768	Facet joint osteoarthritis	unconfirmed
2	hsa_circ_0000893	Breast cancer	28744405
3	hsa_circ_0046264	Coronary artery disease	unconfirmed
4	hsa_circ_0039353	Bladder cancer	unconfirmed
5	hsa_circ_0071896	Facet joint osteoarthritis	29470979
6	hsa_circ_0001112	Colorectal cancer	unconfirmed
7	hsa_circ_0087537	Facet joint osteoarthritis	29470979
8	circVRK1	Breast cancer	29221160
9	hsa_circ_0003570	basal cell cancer	unconfirmed
10	hsa_circ_0020397	Colorectal cancer	28707774
11	hsa_circ_0011316	Colorectal cancer	unconfirmed
12	hsa_circ_0098964	Coronary artery disease	28045102
13	hsa_circ_0051172	Coronary artery disease	28947970
14	hsa_circ_0000069	Colorectal cancer	28003761
15	hsa_circ_0078768	Active pulmonary tuberculosis	28846924
16	hsa_circ_0003838	Breast cancer	28803498
17	hsa_circ_0007006	Colorectal cancer	28656150
18	circRPAP2	Cutaneous squamous cell cancer	unconfirmed
19	hsa_circ_0058792	Coronary artery disease	unconfirmed
20	hsa_circ_0001667	Breast cancer	28803498
21	hsa_circ_0088452	Active pulmonary tuberculosis	28846924
22	hsa_circ_0001087	breast cancer	unconfirmed
23	hsa_circ_0002874	Breast cancer	28803498
24	circUGP2_2	Cervical cancer	unconfirmed
25	circC3	Facet joint osteoarthritis	unconfirmed
26	hsa_circ_0089378	Coronary artery disease	unconfirmed
27	hsa_circRNA_104333	Basal cell cancer	unconfirmed
28	hsa_circ_0002495	Bladder cancer	29558461
29	hsa_circ_0001721	Breast cancer	28744405
30	hsa_circ_0000745	Gastric cancer	28974900

### Performance on independent data set

The results indicate that this method is reliable for circRNA-disease association prediction. In order to further support this conclusion, we verified the method in other databases (CRDD, circRNADisease, and Circ2Disease). It is not possible to identify all potential circRNA disease associations because each database is incomplete. So, we assume that the associations in the database are the only known associations that have been experimentally verified, and the rest are set to unknown associations. The training samples and test samples are described as follows:

$$\left\{ \begin{array}{l} {Z_{1}}_{circR2Disease}^{train}=Z_{1}\\ {Z_{1}}_{circR2Disease}^{test}=C_{U}Z_{1}\cap Z_{database} \end{array}\right.$$ (33)

where ${Z_{1}}_{database}^{train}$ and ${Z_{1}}_{database}^{test}$ are the training set and test set of the independent data sets respectively. *Z*_*database*_ represents the independent data sets, such as CRDD, circRNADisease, and Circ2Disease. In this experiment, the iCDA-CGR was utilized to construct the prediction model using the base dataset *Z*_1_. Since the disease and circRNA are different for each data source, the intersection of all possible association sets C_*U*_*Z*_1_ with independent data set *Z*_*database*_ is used as the test set ${Z_{1}}_{circR2Disease}^{test}$. It can be seen from Table 8 that the proposed method obtained predicted values of 95.18% (Circ2Disease), 90.64% (circRNADisease) and 95.89% (CRDD) in three databases, respectively. In addition, we did the same on circFunBase. The training samples and test samples are described as follows:

$$\left\{ \begin{array}{l} {Z_{2}}_{circR2Disease}^{train}=Z_{2}\\ {Z_{2}}_{circR2Disease}^{test}=C_{U}Z_{2}\cap Z_{database} \end{array}\right.$$ (34)

It can be seen from Table 8 that the proposed method obtained predicted values of 63.26% (Circ2Disease), 73.43% (circRNADisease) and 72.72% (CRDD) in three databases, respectively. The experiment shows that the iCDA-CGR has strong generalization ability.

**Table 8:** Predictive results of the iCDA-CGR on other three databases.

Benchmark dataset	Database	Test pairs	True pairs	Accuracy (%)
circR2disease	Circ2Disease	83	79	95.18
	circRNADisease	171	155	90.64
	CRDD^a^	438	420	95.89
circFunBase	Circ2Disease	49	31	63.26
	circRNADisease	128	94	73.43
	CRDD^a^	121	88	72.72

^a^ website: http://chengroup.cumt.edu.cn/CRDD/

## Discussion

In this study, we proposed the calculation model iCDA-CGR based on quantify location and non-linear information to identify the circRNA-disease associations. This model integrates circRNA sequence information, gene-circRNA associations information, circRNA-disease associations information and the disease semantic information, and predicts the final results by SVM classifier. In particular, we introduce circRNA sequence information and extract the biological sequence position information and quantifies the biological sequence nonlinear relationship of circRNA by Chaos Game Representation for the first time in the circRNA-disease prediction model. The model achieved outstanding results in the experiments of five cross-validation, comparisons with other methods, and independent data sets. Furthermore, 19 of the top 30 circRNA-disease associations predicted in case studies experiments were confirmed by the latest published literature. Due to the addition of sequence information, iCDA-CGR exhibited strong reliability and stability in predicting potential circRNA-disease associations. These experimental results indicate that the sequence information has sufficient coverage relative to nucleic acids, and iCDA-CGR has great potential for nucleic acid function analysis.

## Supporting information

S1 Table Data distribution of the benchmark set circR2Disease and circFunBase of circRNA-disease association. (XLSX)

S2 Table Known circRNA-disease associations obtained from circR2Disease database. (XLSX)

S3 Table Names of 533 circRNAs involved in known circRNA-disease associations obtained from circR2Disease database. (XLSX)

S4 Table Names of 89 diseases involved in known circRNA-disease associations obtained from circR2Disease database. (XLSX)

S5 Table The final disease similarity matrix. (XLSX)

S6 Table The final circRNA similarity matrix. (XLSX)
